# Essential role of the nuclear isoform of *RBFOX1*, a candidate gene for autism spectrum disorders, in the brain development

**DOI:** 10.1038/srep30805

**Published:** 2016-08-02

**Authors:** Nanako Hamada, Hidenori Ito, Takuma Nishijo, Ikuko Iwamoto, Rika Morishita, Hidenori Tabata, Toshihiko Momiyama, Koh-Ichi Nagata

**Affiliations:** 1 Department of Molecular Neurobiology, Institute for Developmental Research, Aichi Human Service Center, Kasugai, Japan; 2 Japan Society for the Promotion of Science, Tokyo, Japan.; 3 Department of Pharmacology, Jikei University School of Medicine, Tokyo, Japan; 4 Department of Neurochemistry, Nagoya University Graduate School of Medicine, Nagoya, Japan

## Abstract

Gene abnormalities in *RBFOX1,* encoding an mRNA-splicing factor, have been shown to cause autism spectrum disorder and other neurodevelopmental disorders. Since pathophysiological significance of the dominant nuclear isoform in neurons, *RBFOX1*-isoform1 (iso1), remains to be elucidated, we performed comprehensive analyses of *Rbfox1*-iso1 during mouse corticogenesis. Knockdown of *Rbfox1*-iso1 by *in utero* electroporation caused abnormal neuronal positioning during corticogenesis, which was attributed to impaired migration. The defects were found to occur during radial migration and terminal translocation, perhaps due to impaired nucleokinesis. Axon extension and dendritic arborization were also suppressed *in vivo* in *Rbfox1*-iso1-deficient cortical neurons. In addition, electrophysiology experiments revealed significant defects in the membrane and synaptic properties of the deficient neurons. Aberrant morphology was further confirmed by *in vitro* analyses; *Rbfox1*-iso1-konckdown in hippocampal neurons resulted in the reduction of primary axon length, total length of dendrites, spine density and mature spine number. Taken together, this study shows that *Rbfox1*-iso1 plays an important role in neuronal migration and synapse network formation during corticogenesis. Defects in these critical processes may induce structural and functional defects in cortical neurons, and consequently contribute to the pathophysiology of neurodevelopmental disorders with *RBFOX1* abnormalities.

*RBFOX1*, also known as Ataxin-2-binding protein 1 (A2BP1) or FOX1, was first identified as an interacting partner for ATAXIN-2^1^, and is expressed in neuronal tissues as well as muscle and heart^2^,^3^. By binding to the (U)GCAUG element in mRNA precursors^2^,^3^,^4^,^5^,^6^, *RBFOX1* has been reported to play a pivotal role in alternative splicing of genes critical for neuronal development^4^,^5^,^6^,^7^.

Accumulating evidence strongly suggests a role of *RBFOX1* in the etiology of autism spectrum disorder (ASD). Array comparative genomic hybridization (aCGH) and genome-wide linkage studies (GWAS) have demonstrated that *RBFOX1* is associated with autism ASD^8^,^9^,^10^,^11^,^12^,^13^, and chromosome region 16p13, where *RBFOX1* is located, was identified as the location of ASD-implicated genes^14^,^15^. In addition, *RBFOX1* target transcripts predicted by bioinformatic methods significantly overlap with genes implicated in ASD^7^,^16^. Furthermore, using a sophisticated system biology approach (weighed gene co-expression network analysis), *RBFOX1* was found to serve as a “hub” in ASD-gene transcriptome networks^16^. Notably, reduced expression of *RBFOX1* in a subset of ASD patient brains was shown to correlate with altered splicing of its predicted target exons^16^,^17^, and massive splicing changes were detected in 48 ASD-susceptibility genes in ASD patient brains where downregulation of *RBFOX1* was supposed^17^,^18^.

ASD-implicated genes are generally associated with other neurodevelopmental and neuropsychiatric disorders, and none of them are specific for ASD. The same is true of *RBFOX1*. For instance, gene abnormalities in *RBFOX1* have also been associated with intellectual disability (ID) with epilepsy^18^, attention deficit hyperactivity disorder (ADHD)^19^ and schizophrenia^20^,^21^. Therefore, common pathophysiological mechanism(s) mediated by *RBFOX1* abnormalities may underlie the clinical outcome of the aforementioned disorders, although unidentified modifiers might contribute to their clinical complexity. To better understand the pathophysiological basis of ASD and other neurodevelopmental disorders, elucidating the physiological function(s) of *RBFOX1* in the cortical development is essential. While we have recently analyzed the pathophysiological relevance of the minor neuronal cytoplasmic isoform, *Rbfox1*-isoform5 (A2BP1-A030), which was referred to as *Rbfox1*-iso2^22^, the pathophysiological significance of the dominant neuronal nuclear isoform, *Rbfox1*-isoform1 (A2BP1-A016; *Rbfox1*-iso1), remains to be clarified. Thus, we here carried out comprehensive analyses of *Rbfox1*-iso1 to elucidate its role in neurodevelopmental disorders.

## Methods

### Study approval

We followed the Fundamental Guidelines for Proper Conduct of Animal Experiments and Related Activity in Academic Research Institution under the jurisdiction of the Ministry of Education, Culture, Sports, Science and Technology, and all of the protocols for animal handling and treatment were reviewed and approved by the Animal Care and Use Committee of Institute for Developmental Research, Aichi Human Service Center (Approval number, M10).

### Plasmid construction

To avoid confusion, *Rbfox1* isoforms used here are termed accoding to the UniProt website (http://www.uniprot.org/uniprot/Q9JJ43). cDNAs of mouse (m) *Rbfox1*-isoform1 (A2BP1-A016, *Rbfox1*-iso1) (GenBank accession number: AY659954, 396 amino acids (aa)), m*Rbfox1*-isoform5 (A2BP1-A030, m*Rbfox1*-iso2 in our previous study^22^) (GenBank accession number: AY659955, 373 aa), mRbfox2-variant5 (GenBank: NM_001110829)^23^,^24^ and mRbfox3-variant1 (GenBank: NM_001039167) in pCS-MT vector with Myc-tag were kindly provided from Dr. S. Kawamoto (NIH, MD)^23^,^24^. These cDNAs were cloned into a pCAG-Myc vector (Addgene Inc., Cambridge, MA). pCAG-PACKmKO1 was kindly supplied by Dr. F. Matsuzaki (RIKEN, Kobe, Japan) for visualization of the centrosome^25^. pCAG-histone 2B(H2B)–EGFP was used to label chromosomes. pCAG-M-Cre was from Dr S. Miyagawa (Univ. Osaka, Japan)^26^ and pCALNL(loxP-neomycin-loxP)-RFP was made from pCALNL-DsRed (Addgene Inc., Cambridge, MA). pβAct-EGFP was kindly provided by Dr S. Okabe (Univ. Tokyo, Japan)^27^. The following target sequences were inserted into pSuper-puro RNAi vector (OligoEngine, Seattle, WA): m*Rbfox1*-iso1#1, GACTAGGAGCCATGCTGAT (1098–1116 in m*Rbfox1*-iso1); m*Rbfox1*-iso1#2, GTAAAATCGAGGTTAATAA (548–566 in m*Rbfox1*-iso1) (m*Rbfox1*-iso1/2^22^); mRbfox2, GCGACTACATGTCTCTAAT (336–354); mRbfox3, GGAAAATTGAGGTCAATAA (497–515). Numbers indicate the positions from translational start sites. We named these vectors as pSuper-m*Rbfox1*-iso1#1, -m*Rbfox1*-iso1#2, -mRbfox2 and –mRbfox3. All constructs were verified by DNA sequencing. For the control RNAi experiments, we used pSuper-H1.shLuc designed against luciferase (CGTACGCGGAATACTTCGA)^22^. To generate an RNAi-resistant m*Rbfox1*-iso1, m*Rbfox1*-iso1R, silent mutations were introduced, as underlined, in the target sequence (GACCAGATCGCACGCCGAT in m*Rbfox1*-iso1#1).

### Antibodies

Anti-*Rbfox1*(A2BP1) was produced by ourselves as previously described^28^. The following mouse monoclonal antibodies were used; anti-Tau-1 (MAB3420; Chemicon International, Temecula, CA), anti-Myc 9E10 and anti-MAP2 (M4403; Sigma-Aldrich, St Louis, MO). Polyclonal rabbit antibodies used were anti-GFP (#598; MBL, Nagoya, Japan), anti-RFP (#600-401-379; Rockland Immunochemicals, Gilbertsville, PA), anti-Rbfox2 (Fox2) (A300-864A-T, Bethyl Laboratories, Montgomery, TX) and anti-Sept11^29^. Anti-GFP (GFP-1020; Chicken) was purchased from AVES Labs (Tigard, OR).

### Drugs

6-Cyano-7-nitroquinoxalline-2,3-dione (CNQX), D-(−)-2-amino-5-phosphonopentanoic acid (D-AP5) and bicuculline methochloride were purchased from Tocris Bioscience (Bristol, UK). These drugs were stored as frozen stock solutions and dissolved in the perfusing solution just before application in the final concentration indicated.

### Cell culture, transfection, immunofluorescence and western blotting

COS7, mouse primary cortical and hippocampal neurons were cultured essentially as described^30^,^31^. Cells were transfected by Lipofectamine 2000 (Life Technologies Japan, Tokyo) according to the manufacturer’s instructions. Immunofluorescence analyses were done as described^32^. Alexa Fluor 488- or 568-labeled IgG (Life Technologies Japan) was used as a secondary antibody. Fluorescent images were captured using an FV-1000 confocal laser microscope. Quantitative analyses of fluorescent signal intensity were done with ImageJ software. Western blot analyses were conducted and immunoreactive bands were visualized as described^28^. Relative protein level was quantified with NIH Image software based on densitometry.

### *In utero* electroporation

Pregnant ICR mice were purchased from SLC Japan (Shizuoka, Japan). *In utero* electroporation was performed essentially as described^33^. Briefly, 1 μl of nucleotide solution containing expression plasmids and/or pSuper-RNAi plasmid (1 μg each) were introduced with pCAG-EGFP or pCAG-RFP (red fluorescent protein) into the lateral ventricles of embryos, followed by electroporation using CUY21 electroporator (NEPA Gene, Chiba, Japan) with 50 ms of 35 V electronic pulse for 5 times with 450 ms intervals. At least 3 brains were used for each experiment.

### Quantitative analysis of neuronal migration

Distribution of GFP-positive cells in brain slices were quantified as follows. The coronal sections of cerebral cortices containing the labeled cells were classified into 5 bins and the intermediate zone (IZ) as described previously^34^. The number of labeled cells in each region of at least 3 slices per brain was calculated.

### 5-ethynil-2′-deoxyuridine (EdU) incorporation experiments

Embryos were electroporated *in utero* with pCAG-H2B-EGFP vector together with pSuper-H1.shLuc (control) or pSuper-m*Rbfox1*-iso1#1 at E14. Forty h after electroporation, pregnant mice were given an intraperitoneal injection of EdU at 25 mg/kg body weight. One h after injection, brains were fixed with 4% paraformaldehyde and frozen sections were obtained. GFP and EdU were detected with anti-GFP and Alexa Fluor555 azide (Life Technologies Japan), respectively, according to the manufacturer’s protocols.

### Time-lapse imaging

After *in utero* electroporation, organotypic coronal slices (250 μm thick) from the interventricular foramen were prepared with a microtome, placed on an insert membrane (pore size, 0.4 μm; Millipore, Bedford, MA), mounted in agarose gel and cultured. The dishes were then mounted in an incubator chamber (5% CO2 and 40%O2, at 37 °C) fitted onto an FV1000 confocal laser microscope (Olympus, Tokyo, Japan), and the primary somatosensory cortex was examined as described^35^. Approximately 8–15 optical Z sections were acquired automatically every 8 to 15 min for 24 h, and about 10 focal planes (~50 μm-thickness) were merged to visualize the entire shape of the cells.

### Quantitative analysis of axon growth

For estimation of axon growth, RFP signal intensity of the callosal axons was measured in a 170 × 150 μm rectangle on both the ipsilateral (before entering the corpus callosum (CC)) and contralateral (after leaving the CC) sides at the positions indicated. The ratio of the axonal RFP signals in the contralateral side to the corresponding ipsilateral side was calculated using Adobe Photoshop software.

### Quantitative analysis of spine morphologies *in vitro*

Transfected neurons were visualized by immunostaining of GFP and chosen randomly. Images were obtained using an FV-1000 confocal microscope. We usually took 0.5 μm-z series stacks to generate image projections for quantitative analysis. To analyze spine morphology, 150–250 spines (from 16–21 neurons) were measured for each condition. For the analysis of spine density, spines were defined as 0.5–6 μm-length, with or without a head, and measured by counting the number of protrusions at 10 μm-length of primary dendrites. Spine density was first averaged per neuron and means from multiple individual neurons were calculated. Morphological assessments of spine density and shape were conducted blindly.

### Slice preparation for electrophysiology

Mice were killed at postnatal days (P)4 or 7 by decapitation under deep isoflurane anaesthesia, and coronal slices were cut (300 μm-thickness) using a microslicer (PRO7, Dosaka, Kyoto, Japan) in ice-cold oxygenated cutting Krebs solution of the following composition (mM): choline chloride, 120; KCl, 2.5; NaHCO_3_, 26; NaH_2_PO_4_, 1.25; D-glucose, 15; ascorbic acid, 1.3; CaCl_2_, 0.5; MgCl_2_, 7. The slices were then transferred to a holding chamber containing standard Krebs solution of the following composition (mM): NaCl, 124; KCl, 3; NaHCO_3_, 26; NaH_2_PO_4_, 1; CaCl_2_, 2.4; MgCl_2_, 1.2; D-glucose, 10; pH 7.4 when bubbled with 95% O_2_–5% CO_2_. Slices were incubated in the holding chamber at room temperature (21–26 °C) for at least 1 hour before recording.

### Whole-cell recording and data analysis

For recoding, a slice was transferred to the recording chamber, held submerged, and superfused with standard Krebs solution (bubbled with 95% O_2_–5% CO_2_) at a rate of 3–4 ml/min. Neurons in layer II of the cortex were visualized with a 60× water immersion objective attached to an upright microscope (BX50WI, Olympus Optics, Tokyo, Japan). Fluorescent pyramidal neurons were visualized using the appropriate fluorescence filter (U-MWIG3, Olympus). Images were captured with a cooled CCD camera (CCD-300 T-RC, Nippon roper, Tokyo, Japan) and displayed on a video monitor. Patch pipettes for whole-cell recording were made from standard-walled borosilicate glass capillaries (Clark Electromedical, Reading, UK). For the recording of spontaneous or evoked synaptic currents, patch pipettes were filled with a cesium chloride-based internal solution of the following composition (mM): CsCl, 140; NaCl, 9; Cs-EGTA, 1; Cs-HEPES, 10; Mg-ATP, 2. For the recording of membrane potentials, a K-gluconate-based internal solution of the following composition (mM) was used: K-gluconate, 120; NaCl, 6; CaCl_2_, 5; MgCl_2_, 2; K-EGTA, 0.2; K-HEPES, 10; Mg-ATP, 2; Na-GTP, 0.3. Whole-cell recordings were made from fluorescent pyramidal neurons using a patch-clamp amplifier (Axopatch 200B, Molecular Devices, Foster City, CA). The cell capacitance and the series resistance were measured from the amplifier. The access resistance was monitored by measuring capacitative transients obtained in response to a hyperpolarizing voltage step (5 mV, 25 ms) from a holding potential of −65 mV. No correction was made for the liquid junction potentials (calculated to be 5.0 mV by pCLAMP7 software, Molecular Devices). Synaptic currents were evoked at a rate of 0.2 Hz (every 5 s) by extracellularly delivered voltage pulses (0.2–0.4 ms in duration) of suprathreshold intensity via a stimulating electrode filled with 1 M NaCl. The stimulating electrode was placed within 50–120 μm radius of the recorded neuron. The position of the stimulating electrode was varied until a stable response was evoked in the recorded neuron. Experiments were carried out at room temperature.

Data were stored on digital audio tapes using a DAT recorder (DC to 10 kHz; Sony, Tokyo, Japan). Evoked EPSCs were digitized off-line at 10 kHz (low-pass filtered at 2 kHz with an 8-pole Bessel filter) using pCLAMP9 software (Molecular Devices). The effects of drugs on the evoked IPSCs were assessed by averaging their amplitudes for 100 s (20 traces) after the effect had reached the steady state and comparing this value with the averaged amplitude of 20 traces just before the drug application. Spontaneous EPSCs (sEPSCs) or sIPSCs were filtered at 2 kHz and digitized at 20 kHz using pCLAMP9 software and analyzed using N software (provided by Dr. S. F. Traynelis, Emory University).

## Results

### Roles of *Rbfox1*-iso1 in neuronal positioning during corticogenesis

**Figure 1:**
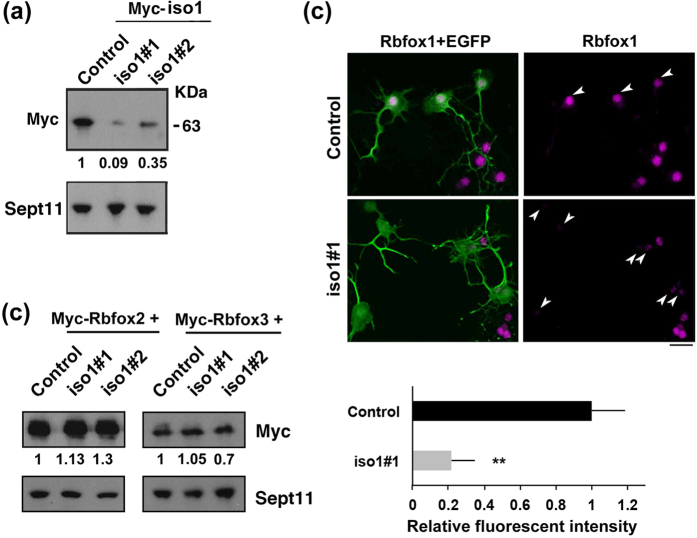
Characterization of pSuper-m*Rbfox1*-iso1 vectors. **(a)** Knockdown of exogenous *Rbfox1*-iso1 in COS7. pCS-MT-m*Rbfox1*-iso1 (Myc-iso1) was cotransfected into COS7 cells with pSuper-H1.shLuc (Control), pSuper-m*Rbfox1*-iso1#1 or -iso#2. After 48 h, cells were harvested and subjected to western blotting with anti-Myc (*upper* panel). Anti-Sept11 was used for loading control (*lower* panel). Relative band intensity was also shown. **(b)** Knockdown of endogenous *Rbfox1*-iso1 in neurons. pCAG-EGFP was transfected with pSuper-H1.shLuc (Control) or pSuper-m*Rbfox1*-iso1#1 into dissociated mouse hippocampal neurons obtained at E16, and cultured *in vitro* for 72 h. After fixation, cells were immunostained with anti-GFP (chicken; green) and anti-*Rbfox1* (magenta). Scale bar shows 10 μm. Quantification of *Rbfox1* expression was performed with ImageJ by analyzing the nuclei of control and deficient cells (arrowheads). **(c)** Effects of m*Rbfox1*-iso1-knockdown on expression of Rbfox2 and Rbfox3. pCAG-Myc-mRbfox2 or –mRbfox3 was cotransfected into COS7 cells with pSuper-H1.shLuc (Control), pSuper-m*Rbfox1*-iso1#1 or -iso#2. Analyses were done as in (**a**).

Since neuronal migration is essential for corticogenesis, we examined the role of *Rbfox1*-iso1 in the migration of newly generated cortical neurons by RNAi experiments. We first confirmed that pSuper-m*Rbfox1*-iso1#1 efficiently knocked down exogenous mouse (m)*Rbfox1*-iso1 in COS7 cells and endogenous *Rbfox1*-iso1 in primary cultured mouse hippocampal neurons (Fig. 1a,b). It should be noted here that we could not prepare another RNAi vector specific for m*Rbfox1*-iso1, since *Rbfox1*-iso1 is identical to *Rbfox1*-iso5 except for the C-terminal 66 aa. We thus had to use pSuper-m*Rbfox1*-iso1/2^22^, which targets a common sequence with *Rbfox1*-iso5, as the second RNAi vector. Notably, neither pSuper-m*Rbfox1*-iso1#1 nor –iso1#2 silenced *Rbfox1* homologous proteins, mRbfox2 and mRbfox3, in COS7 cells, indicating the specificity of these RNAi vectors (Fig. 1c).

**Figure 2:**
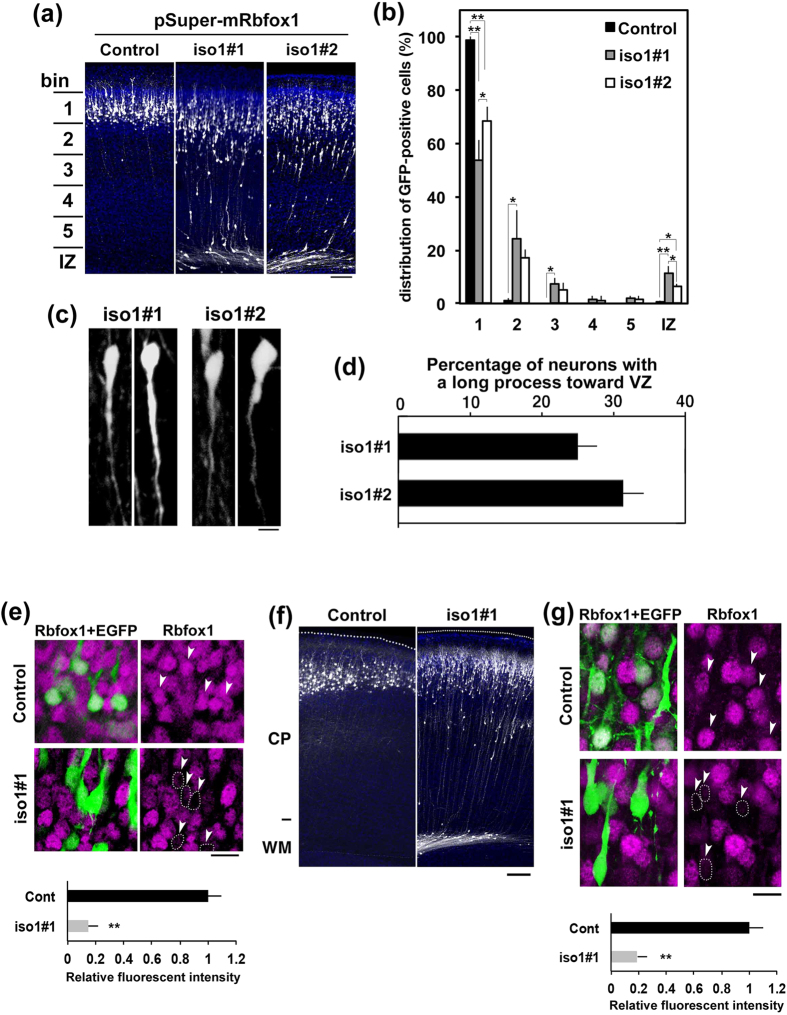
Role of *Rbfox1*-1 in cortical neuron migration during mouse brain development. **(a)** Migration defects of *Rbfox1*-deficient cortical neurons. pCAG-EGFP was coelectroporated with pSuper-H1.shLuc (Control), pSuper-m*Rbfox1*-iso1#1 or -iso1#2 into cerebral cortices at E14.5. Coronal sections were prepared at P3 and immunostained with anti-GFP (white) and DAPI (blue). Scale bars in (**a,f**), 100 μm. **(b**) Quantification of the distribution of the deficient neurons in distinct parts of the cerebral cortex (bin 1–5, and IZ) for each condition shown in (**a**) Error bars indicate SD (n = 3); **p < 0.01, *p < 0.05 by Tukey-Kramer LSD. (**c)** Representative images of *Rbfox1*-iso1-deficient neurons remained in CP at P3 under the condition in (**a**) Scale bar, 5 μm. (**d**) Quantification of the cortical neurons with a long process toward VZ. Numbers of cells used for each calculation were more than 150 in each condition in (**c**). Error bars indicate SD. Note that control results are not shown since the control neurons did not show migration delay. **(e**) Knockdown of *Rbfox1*-iso1 *in vivo* in neurons with abnormal positioning. Coronal sections prepared at P3 as in (**a**) were stained for GFP (green) and *Rbfox1* (magenta). Arrowheads indicate GFP-positive cells near the pial surface (Control) or in CP (iso1#1). *Rbfox1*-deficient cells were encircled by dotted line. Quantification of *Rbfox1* expression was performed with ImageJ by analyzing the fluorescent intensity in the control and deficient cells (arrowheads). Scale bars in (**e**,**g**), 10 μm. **(f)** Positional defects of the deficient neurons at P7. *In utero* transfection was done and coronal sections were immunostained as in (**a**). **(g)** Knockdown of *Rbfox1*-iso1 *in vivo* at P7. Coronal sections were stained as in (**e**) Arrowheads indicate GFP-positive cells. Quantification of *Rbfox1* expression was performed as in (**e**).

pCAG-EGFP was coelectroporated with pSuper-H1.shLuc (control) or pSuper-m*Rbfox1*-iso1-RNAi vectors into progenitor and stem cells lining the ventricular zone (VZ) of embryonic day (E)14.5 mice brains by *in utero* electroporation. When harvesting and analysis at P3, it was found that control neurons were located in the superficial layers (bin 1; layers II∼III) of the cortical plate (CP) (Fig. 2a, *Control* panel, and B). In contrast, *Rbfox1*-iso1-deficient neurons were abnormally distributed in the lower zone of the CP and intermediate zone (IZ) (Fig. 2a, *iso1#1* and *#2* panels, and B). Since cell morphology is closely associated with cell migration, we examined the shape of the deficient neurons with abnormal positioning in Fig. 2a. The deficient neurons frequently had a long process extending toward the VZ although these cells maintained bipolar morphology (Fig. 2c,d), suggesting that *Rbfox1*-iso1 may regulate cortical neuron morphology. We confirmed the knockdown of *Rbfox1*-iso1 in cortical neurons with migration defects by performing immunohistochemical staining (Fig. 2e). Analysis of cortical migration at a later time point (P7) again demonstrated a migration delay with many *Rbfox1*-deficient cells failing to reach their target destination (layers II–III) (Fig. 2f,g).

**Figure 3:**
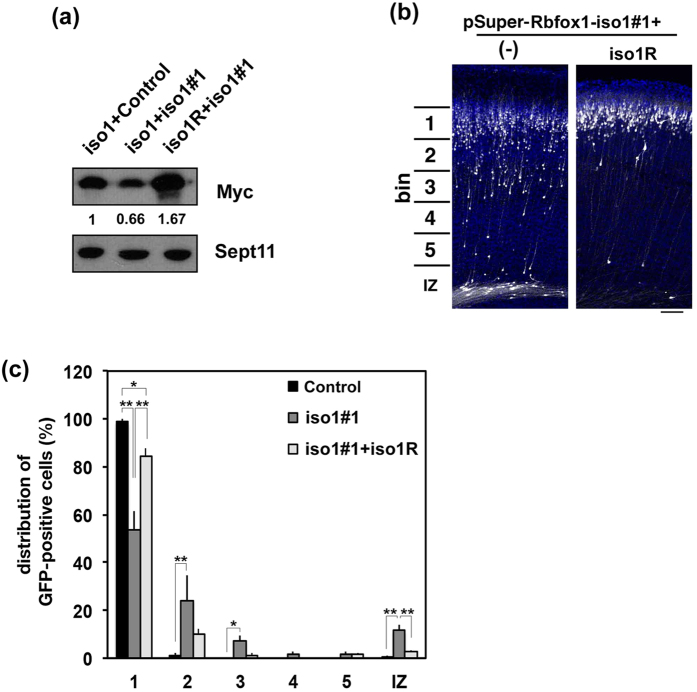
Rescue of *Rbfox1*-iso1-knockdown-induced migration defects. **(a)** Characterization of an RNAi-resistant version of *Rbfox1*, m*Rbfox1*-iso1R. pCAG-Myc-m*Rbfox1*-iso1 (iso1) or –iso1R was cotransfected into COS7 cells with pSuper-H1.shLuc (Control) or pSuper-m*Rbfox1*-iso1#1. After 48 h, cells were harvested and subjected to western blotting with anti-Myc. Anti-Sept11 was used for loading control. Relative band intensity was also shown. **(b**) pCAG-EGFP was coelectroporated with pSuper-m*Rbfox1*-iso1#1 together with pCAG vector (−) or pCAG-Myc-m*Rbfox1*-iso1R into cerebral cortices at E14.5. After fixation at P3, analyses were performed as in Fig. 2a. Scale bar, 100 μm. (**c**) Quantification of the distribution of GFP-positive neurons in distinct parts of the cerebral cortex (bin 1–5, and IZ) for each condition shown in (**b**). Error bars indicate SD (n = 3); **p < 0.01, *p < 0.05 by Tukey-Kramer LSD.

Rescue experiments were then performed to rule out off-target effects. To this end, we used m*Rbfox1*-iso1R that was resistant to pSuper-m*Rbfox1*-iso1#1-mediated silencing (Fig. 3a). When pSuper-m*Rbfox1*-iso1#1 was coelectroporated with pCAG-Myc-m*Rbfox1*-iso1R, positional defects were rescued at P3 (Fig. 3b,c), indicating that the abnormal positioning observed was indeed caused by reduction of *Rbfox1*-iso1 expression.

**Figure 4:**
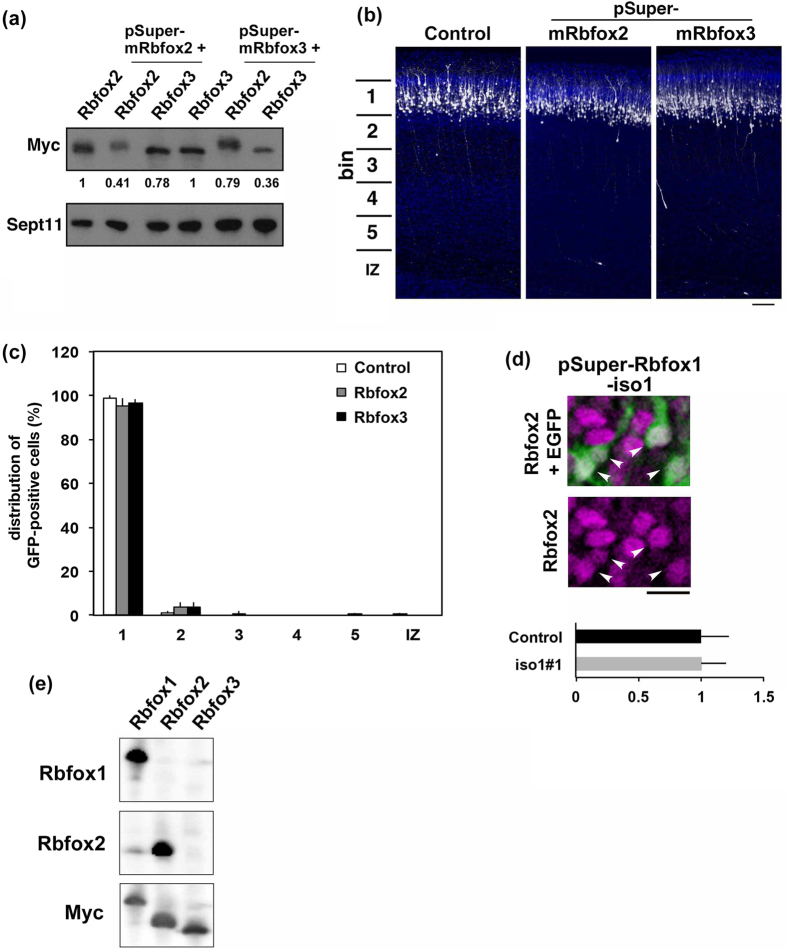
Role of Rbfox2 and Rbfox3 in neuronal migration during mouse brain development. **(a)** Characterization of pSuper-mRbfox2 and –mRbfox3 vectors. pCS-MT-mRbfox2 or –mRbfox3 was cotransfected into COS7 cells with pSuper-mRbfox2 or –mRbfox3 in various combinations. After 48 h, cells were harvested and subjected to western blotting with anti-Myc. Anti-Sept11 was used for a loading control. Relative band intensity was also shown. **(b)** Knockdown of Rbfox2 or Rbfox3 in migrating neurons. pCAG-EGFP was coelectroporated with pSuper-H1.shLuc (Control), pSuper-mRbfox2 or -mRbfox3 into cerebral cortices at E14.5 and fixed at P3. Coronal sections were immunostained with anti-GFP (white) and DAPI (blue). Scale bar, 100 μm. **(c**) Quantification of the distribution of Rbfox2- or Rbfox3-deficient neurons in distinct parts of the cerebral cortex (bin 1–5, and IZ) for each condition shown in (**b**). Error bars indicate SD (n = 3). **(d**) Effects of *Rbfox1*-iso1-knockdown on the expression of Rbfox2. A coronal section prepared at P7 as in Fig. 2g was stained for GFP (green) with Rbfox2 (magenta). Double-positive cells for GFP and Rbfox2 were indicated by arrowheads. Quantification of Rbfox2 expression was performed as in Fig. 2e. Note that Rbfox2 expression was comparable to surrounding internal control neurons. Bars, 10 μm. (**e**) Characterization of anti-Rbfox2 antibody. Lysates (20 μg of protein per lane) from COS7 cells transiently expressing Myc-*Rbfox1*-iso1, -Rbfox2 or –Rbfox3 were subjected to western blotting with anti-Rbfox2.

We next examined if Rbfox2 and Rbfox3 are implicated in the cortical neuron positioning since these proteins are highly homologous to *Rbfox1*. pSuper-mRbfox2 and –mRbfox3 efficiently knocked down mRbfox2 and mRbfox3, respectively, in COS7 cells (Fig. 4a). When endogenous Rbfox2 or Rbfox3 was silenced in stem and progenitor cells in VZ at E14.5, the neurons migrated normally to the superficial layer (bin 1; layers II~III) of CP as in the control experiment (Fig. 4b,c). These experiments strongly suggest that Rbfox2 and Rbfox3 are not involved in the positioning of cortical neurons under our experimental conditions. On the other hand, Rbfox2 is crucial for cerebellar development and mature motor function^36^ while gene abnormalities of RBFOX3 contribute to the generalized idiopathic epilepsy syndromes^37^. It remains to be clarified if RBFOX3 and RBFOX2 are involved in the establishment of cortical architecture and neurodevelopmental disorders including ASD.

As it has previously been shown that the expression of Rbfox2 (but not Rbfox3) is increased in the brain of *Rbfox1*-knockout mouse, we analyzed the expression of endogenous Rbfox2 in the *Rbfox1*-iso1-deficient neurons^6^. We found that the expression of endogenous Rbfox2 in cortical neurons was not affected by the acute knockdown of *Rbfox1*-iso1 at P7 (Fig. 4d,e). These results strongly suggest that the abovementioned abnormal phenotypes were due to *Rbfox1*-iso1-silencing and not secondary effects due to changes in Rbfox2 levels.

### *Rbfox1*-iso1 does not regulate neuronal progenitor proliferation

Previous study has shown that cell cycle defects can result in neuronal migration delay^38^. We thus asked if the migration delay in this study was caused by cell cycle delay. To this end, we looked into the effect of *Rbfox1*-iso1-silencing on the cell cycle of stem and progenitor cells in VZ/subventricular zone (SVZ). E14.5 cortices were coelectroporated with pCAG-H2B-EGFP together with pSuper-H1.shLuc (control) or pSuper-m*Rbfox1*-iso1#1. To detect DNA replication, EdU incorporation was done as described in “Materials and Methods”. After coronal sections were visualized for GFP and EdU, the ratio of EdU/GFP double-positive cells among GFP-positive cells was determined (Control, 20.3 ± 2.08 (n = 3); iso1#1, 18.3 ± 0.577(n = 4)). Numbers of cells used for each calculation were more than 100. These results indicate that *Rbfox1*-iso1-deficient cells entered S-phase to a similar extent when compared to control cells and that the rate of G1-progression was not statistically different between control and *Rbfox1*-iso1-deficient cells. We therefore assume that knockdown of *Rbfox1*-iso1 did not affect cell division/proliferation at VZ/SVZ. Since *Rbfox1*-iso1 was not involved in the cell cycle of VZ cells and not expressed in VZ/SVZ^28^, abnormal positioning of cortical neurons by *Rbfox1*-iso1-knockdown was most likely to be caused by migration defects.

### Time-lapse imaging of migration of *Rbfox1*-iso1-deficient neurons in cortical slices

**Figure 5:**
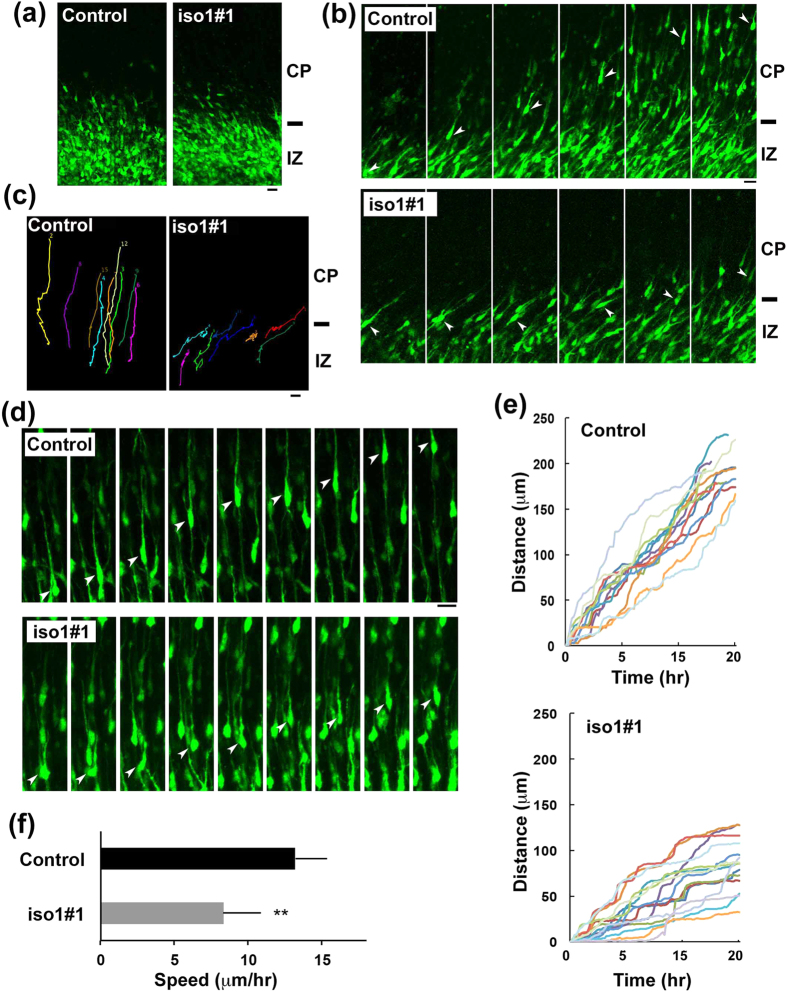
Time-lapse imaging of *Rbfox1*-iso1-deficient neuron migration. **(a)** Cortical slices at the beginning of tissue culture under the confocal microscope were shown. E14.5 cortices were coelectroporated with pCAG-EGFP with pSuper-H1.shLuc (Control) or pSuper-m*Rbfox1*-iso1#1, followed by coronal section slice preparation at E16.5 and time-lapse imaging. There was no difference in transfection efficiency between the experiments. Scale bars in (**a–d**), 20 μm. **(b)** Time-lapse imaging of control and *Rbfox1*-iso1-deficient neurons at IZ-CP boundary. **(c)** Tracing of control or the deficient neurons (iso1#1) in upper IZ - lower CP in (**b**) Migratory tracks of 8 cells were demonstrated as color lines. **(d)** Time-lapse imaging of control and the deficient neurons (iso1#1) migrating in CP. **(e)** Migration distance of control and the deficient neurons (iso1#1) in CP after crossing IZ. Thirteen cells were selected in (**d**) and analyzed. **(f)** Calculation of migration velocity of control and the deficient neurons (iso1#1) in middle CP. Twenty cells were analyzed in each experiment (n = 3). Error bars indicate SD; **p < 0.01 by Student’s *t*-test. Analyses were repeated 3 times for each case. Representative results were shown in (**a–e**).

Newborn cortical neurons are primarily multipolar and exhibit slow and irregular movement in the lower IZ. After a certain period (~24 h), they transform into a bipolar shape with a leading process and an axon in the upper IZ, move into CP and exhibit radial migration toward pial surface^39^,^40^. We performed detailed analyses of *Rbfox1*-iso1 in neuronal migration and morphology in IZ and CP by time-lapse imaging. To this end, VZ cells were coelectroporated with pCAG-EGFP together with the control vector or pSuper-m*Rbfox1*-iso1#1 at E14.5. At the beginning of imaging (E16.5), control and *Rbfox1*-iso1-deficient cells appeared to be multipolar while some cells were transforming into bipolar neurons (Fig. 5a). However, when time-lapse imaging was continued, differences in radial migration were observed between control and the deficient cells. In the control experiments, GFP-positive neurons normally transformed from multipolar to bipolar in the upper IZ, smoothly migrated into CP and then moved toward the pial surface (Fig. 5b,c and Supplementary video 1). In contrast, the deficient cells frequently remained stranded in the upper IZ - lower CP after shape change into bipolar status (Fig. 5b,c and Supplementary video 2). These results suggest that *Rbfox1*-iso1-silencing has no effects on multipolar-bipolar transition and abrogates the initiation of radial migration.

Although some deficient cells appeared to cross IZ in a smooth manner, they frequently showed abnormal migration in the CP. We monitored the migration and morphology of such cells. When compared to control neurons that exhibit normal locomotion toward the pial surface (Fig. 5d,e and Supplementary video 3), *Rbfox1*-deficient cells showed an unusual migration delay in CP; swelling formation and subsequent nucleokinesis (translocation of the nucleus into the leading process) were drastically delayed and cells displayed a characteristic “stepwise” migration phenotype (Fig. 5d,e and Supplementary Video 4). The average migration velocity in the CP was reduced for such cells (Fig. 5f). Collectively, *Rbfox1*-iso1 may regulate two steps of cortical neuron migration; smooth crossing of the IZ-CP border and subsequent radial migration in the CP. While migration defects might occur at the IZ-CP border when the RNAi effect is strong, delayed migration in the CP and defective terminal translocation (see below) might be observed when the RNAi effect is relatively weak. Since bioplar polarity was maintained in the deficient cells during radial migration (E17.5–18.5) (Fig. 5d), we suppose that the upside-down shape observed in Fig. 2c,d was formed at later migration stage or after abnormal positioning.

### *Rbfox1*-iso1 regulates nucleokinesis of migrating cortical neurons during corticogenesis

**Figure 6:**
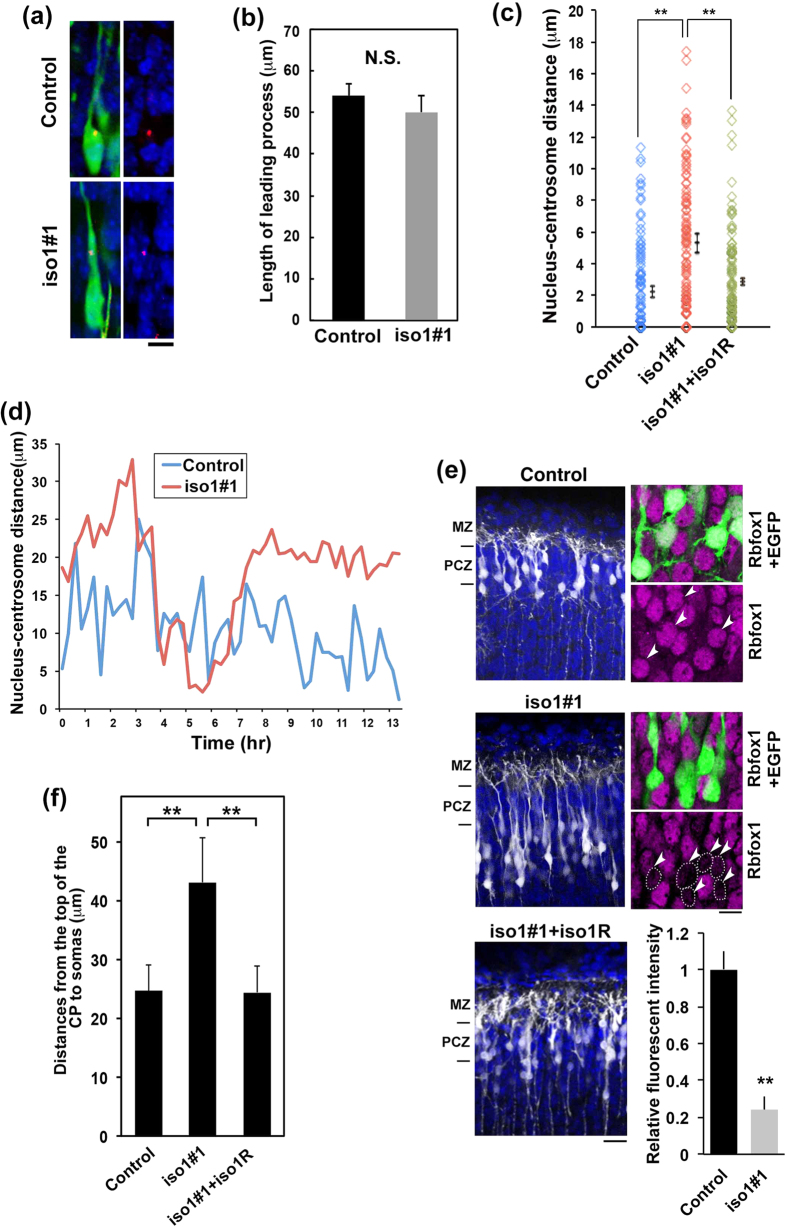
Coupling of the nucleus to centrosome is defective in *Rbfox1*-iso1-deficient neurons. **(a**) Cell shape of control and *Rbfox1*-iso1-deficient neurons during radial migration. pCAG-EGFP was electroporated with pCAG-PACKmKO1 (a marker for centrosome) with pSuper-H1.shLuc or pSuper-m*Rbfox1*-iso1#1 into cerebral cortices at E14.5. GFP, centrosome (red) and DNA (blue) were immunostained in coronal sections at E17.5. Representative images of migrating neurons in lower CP were shown. Scale bar, 10 μm. (**b**) Quantification of the length of leading process of control and *Rbfox1*-iso1-deficient neurons. Numbers of cells for each calculation was more than 50. Error bars indicate SD. (**c**) The N-C distance between centrosome and the top of nucleus was measured. pCAG-EGFP was transfected with pCAG-PACKmKO1 together with pSuper-H1.shLuc (Control), pSuper-m*Rbfox1*-iso1#1 or pSuper-m*Rbfox1*-iso1#1 plus pCAG-Myc-m*Rbfox1*-iso1R into E14.5 mouse brains, and fixed at E17.5. Numbers of cells for each calculation was 100 cells. Error bars indicate SD; **p < 0.01 by Tukey-Kramer LSD (n = 3). (**d**) Time-course profiles of the N-C distance dynamics of control and the deficient neurons (iso1#1). **(e**) Effects of *Rbfox1*-iso1-knockdown for the terminal translocation. Cerebral cortices were electroporated with pCAG-EGFP with pSuper-H1.shLuc (Control), pSuper-m*Rbfox1*-iso1#1 or pSuper-m*Rbfox1*-iso1#1 plus pCAG-Myc-m*Rbfox1*-iso1R at E15.5, and analyzed at P3. MZ, marginal zone; PCZ, primitive cortical zone. *Rbfox1* expression was also analyzed (*right* panels). GFP-positive cells were indicated by arrowheads while *Rbfox1*-deficient cells were encircled by dotted line. Quantification of *Rbfox1* expression was performed for the deficient cells as in Fig. 2e. (**f**) Statistical analyses of (**e**). Distance between the top of CP and the cell soma was measured. Error bars represent SD. **p < 0.01 by Tukey-Kramer LSD (n = 3).

Radial migration is composed of leading process extension and nucleokinesis. Since the leading process appears to form normally in *Rbfox1*-iso1-deficient neurons during radial migration (Fig. 6a,b), the defective “stepwise” migration observed in Fig. 5 may be due to defects in nucleokinesis. Nucleokinesis consists of 1) advancement of the centrosome, the site of microtubule emanation, into a proximal ‘swelling’ in the leading process, and 2) translocation of the nucleus, enveloped in a “cage”-like structure by centrosome-derived microtubules, towards the centrosome^41^. Since the relative position of the centrosome and the nucleus is critical for nucleokinesis^42^, we asked if the coupling of the nucleus to the preceding centrosome is dependent on *Rbfox1*-iso1. To this end, the distance between the nucleus and centrosome (N-C distance) in migrating neurons was measured with cortical slices. Consequently, N-C distance was significantly longer in *Rbfox1*-iso1-deficient neurons (Fig. 6c). Further live-imaging analysis confirmed an abnormally elongated and prolonged N-C distance in the deficient neurons (Fig. 6d and Supplementary videos 5 and 6).

At the end of migration process, the mode changes from radial migration to terminal translocation just beneath the marginal zone (MZ). Terminal translocation is a crucial step for the completion of neuronal migration^43^. Since correct nucleokinesis is also essential for the terminal translocation, we looked into the effects of *Rbfox1*-iso1-knockdown. As shown in Fig. 6e,f, terminal translocation was not completed for *Rbfox1*-iso1-deficient neurons; cells could not enter the outermost region of CP termed the primitive cortical zone (PCZ), although the tip of the process could attach to the MZ. Notably, RNAi-resistant m*Rbfox1*-iso1R rescued the knockdown phenotype (Fig. 6e,f). We assume that the hampered terminal translocation was caused by mild *Rbfox1*-iso1-silencing conditions, where neurons migrated to the cortical surface but could not complete the whole migration process.

The above results indicate that nucleokinesis was hindered in the *Rbfox1*-iso1-deficient cells. Since microtubule organization is crucial for nucleokinesis, disrupted microtubule dynamics is considered to be an underlying mechanism for the migration defects.

### *Rbfox1*-iso1 regulates axon and dendrite development *in vivo*

**Figure 7:**
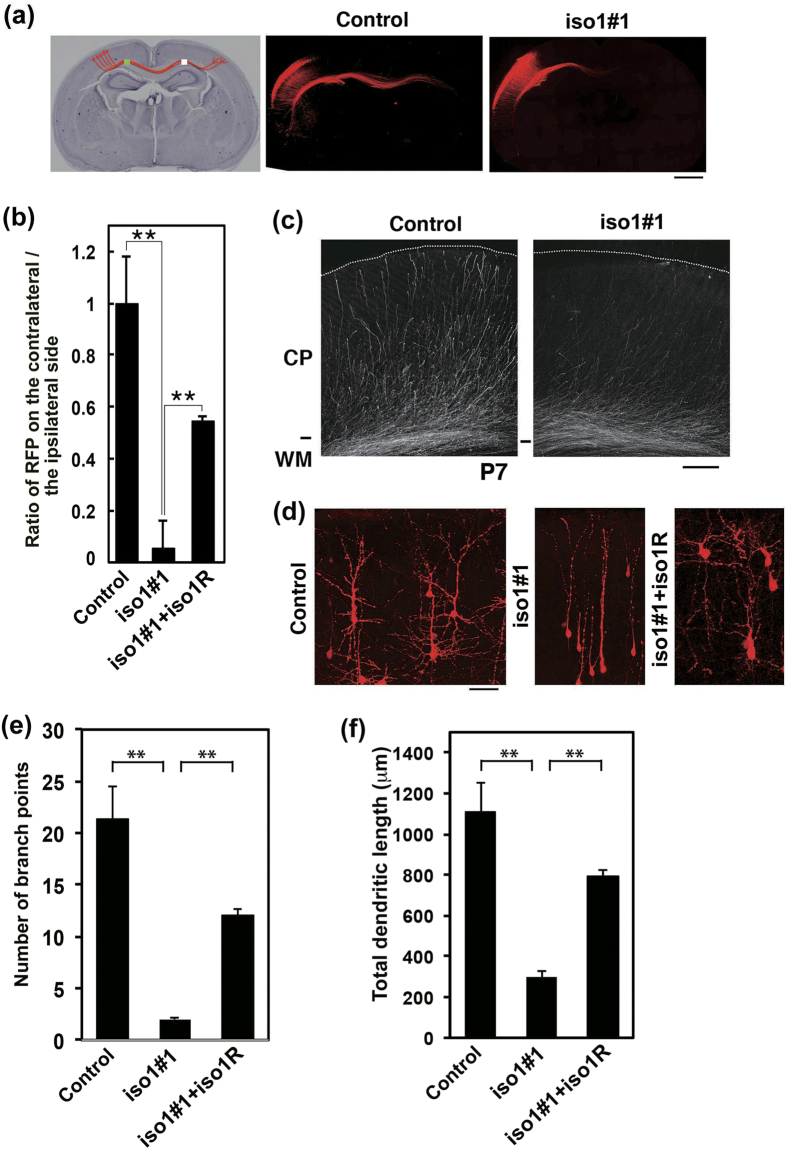
Role of *Rbfox1*-iso1 in the axon and dendrite growth in cortical neurons *in vivo*. **(a)** Effects of *Rbfox1*-iso1-knockdown on the axon elongation. pCAG-RFP was coelectroporated with pSuper-H1.shLuc (Control) or pSuper-m*Rbfox1*-iso1#1 into cerebral cortices at E14.5, and analyzed at P3. Hematoxylin staining of a slice was also shown (*left* panel). Scale bar, 1 mm. **(b)** Quantitative analyses of the ratio of the intensity of RFP-positive axons in the area (white) of the contralateral cortex to that in the area (green) of ipsilateral cortex in (**a**). Rescue experiments were done by cotransfection with pCAG-Myc-m*Rbfox1*-iso1R. Error bars indicate SD; **p < 0.01 by Tukey-Kramer LSD (n = 3). **(c**) Representative images of the terminal arbors of callosal axons expressing RFP with pSuper-H1.shLuc (Control) or pSuper-m*Rbfox1*-iso1#1 at P7. Scale bar, 200 μm. **(d**) *Rbfox1*-iso1-knockdown inhibits dendritic branching *in vivo*. pCAG-loxP-RFP was electroporated for sparse expression with pCAG-M-Cre together with pSuper-H1.shLuc (Control), pSuper-m*Rbfox1*-iso1#1 or pSuper-m*Rbfox1*-iso1#1 plus pCAG-Myc-m*Rbfox1*-iso1R into cerebral cortices at E14.5. Analyses were carried out in cortical slices at P7. Representative average Z-stack projection images of RFP fluorescence of cortical neurons in upper CP were shown. Scale bar, 50 μm. (**e,f**) Number of dendritic branch points (**e**) or total dendritic length (**f**) was calculated on neurons observed in (**d**). Three brains were analyzed for each experiment; Control, n = 18 neurons; pSuper-m*Rbfox1*-iso1#1, n = 22; pSuper-m*Rbfox1*-iso1#1 plus pCAG-Myc-m*Rbfox1*-iso1R, n = 21. Error bars indicate SD; **p < 0.01 by Tukey-Kramer LSD.

Since various neurodevelopmental disorders including ASD are thought to be “synapse” diseases, *Rbfox1*-iso1 should be involved in axon and dendrite network formation. We thus investigated whether *Rbfox1*-iso1-deficiency actually affects axon elongation and dendrite arborization during brain development. When *Rbfox1*-iso1 was silenced in VZ stem and progenitor cells at E14.5 and axons were visualized in the contralateral hemisphere at P3, axon density became lower after leaving the corpus callosum (Fig. 7a,b). The phenotype was at least partially rescued by m*Rbfox1*-iso1R (Fig. 7b). Although axons from the hemisphere containing the deficient cells reached efficiently the contralateral white matter at P7, such axons did not extend properly into the cortical layers on the contralateral side (Fig. 7c). These results strongly suggest that *Rbfox1*-iso1 is involved in the axon elongation of cortical neurons.

We next examined the role of *Rbfox1*-iso1 in dendritic arbor formation. Introduction of pSuper-m*Rbfox1*-iso1#1 at E14.5 into VZ cells resulted in highly abrogated dendritic arborization at P7 (Fig. 7d). Branch point number and total length of dendrites were significantly decreased in the deficient neurons compared to those in matching wild-type cells (Fig. 7e,f), suggesting that *Rbfox1*-iso1 participates in dendrite formation and maintenance. These abnormal phenotypes were rescued at least partially by m*Rbfox1*-iso1R (Fig. 7e,f).

Taken together, the functional loss of *Rbfox1*-iso1 may impair synaptic connectivity through defects in axon and dendrite network formation. The clinical symptoms of ASD and other neurodevelopmental disorders with *RBFOX1* gene abnormalities may reflect the observed cellular phenotypes.

### Role of *Rbfox1*-iso1 in neuronal morphology *in vitro*

**Figure 8:**
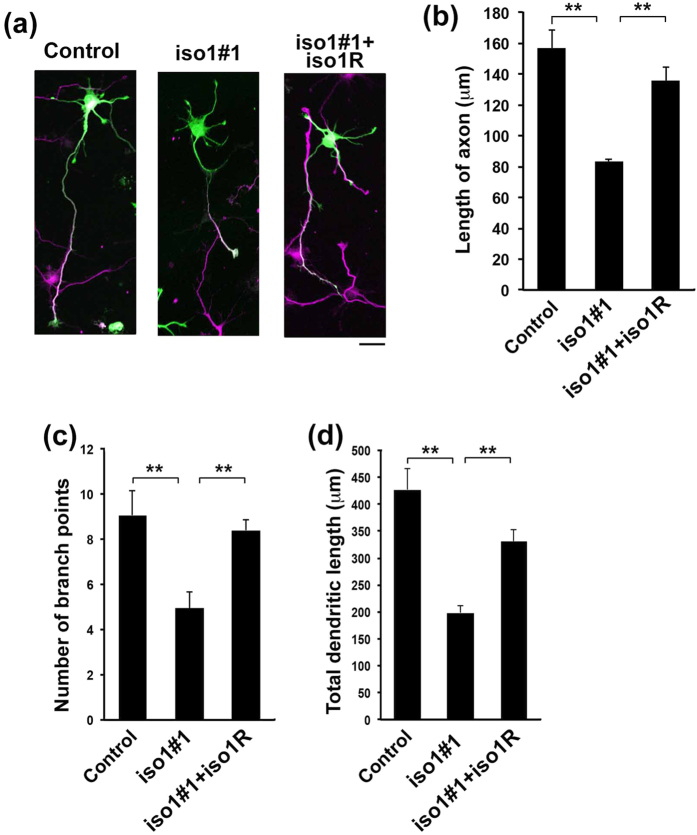
Role of *Rbfox1*-iso1 in axon and dendrite development of primary cultured hippocampal neurons. **(a)** Dissociated neurons were obtained from E16 mice, followed by cotransfection of pCAG-EGFP with pSuper-H1.shLuc (Control), pSuper-m*Rbfox1*-iso1#1 or pSuper-m*Rbfox1*-iso1#1 plus pCAG-Myc-m*Rbfox1*-iso1R. After 72 hr, cells were fixed and immunostained with anti-GFP (green) and anti-tau-1 (magenta). Scale bars, 20 μm. **(b)** Quantification of the length of primary axon. Numbers of cells used for each calculation was more than 100. Error bars indicate SD (n = 3); **p < 0.01 by Tukey-Kramer LSD. (**c,d**) *Rbfox1*-iso1-knockdown inhibits dendritic growth. pβAct-GFP was electroporated with pSuper-H1.shLuc (Control), pSuper-m*Rbfox1*-iso1#1 or pSuper-m*Rbfox1*-iso1#1 plus pCAG-Myc-m*Rbfox1*-iso1R into dissociated neurons at 0 div. After cells were fixed and stained for GFP (green) and MAP2 (red) at 7 div, number of dendritic branch points (**c**) or total dendritic length (**d**). Numbers of cells used for each calculation was more than 50. Error bars indicate SD (n = 3); **p < 0.01 by Tukey-Kramer LSD.

Since *Rbfox1*-iso1 was most likely to regulate the development of axon and dendrites *in vivo*, we further clarified if the observed phenomena are cell-autonomous or not by *in vitro* analyses. Knockdown of *Rbfox1*-iso1 was conducted in primary cultured mouse hippocampal neurons, which exhibit highly polarized morphology^44^. The knockdown resulted in a reduction of primary axon length, which was rescued by m*Rbfox1*-iso1R (Fig. 8a,b). On the other hand, single primary axons were observed in both control and *Rbfox1*-iso1-deficient neurons (Fig. 8a). As for the dendrite extension, the branch point number and total length of dendrites in the deficient neurons were both significantly decreased (Fig. 8c,d). These phenotypes were again rescued by m*Rbfox1*-iso1R (Fig. 8c,d).

### Involvement of *Rbfox1*-iso1 in spine morphology *in vitro*

**Figure 9:**
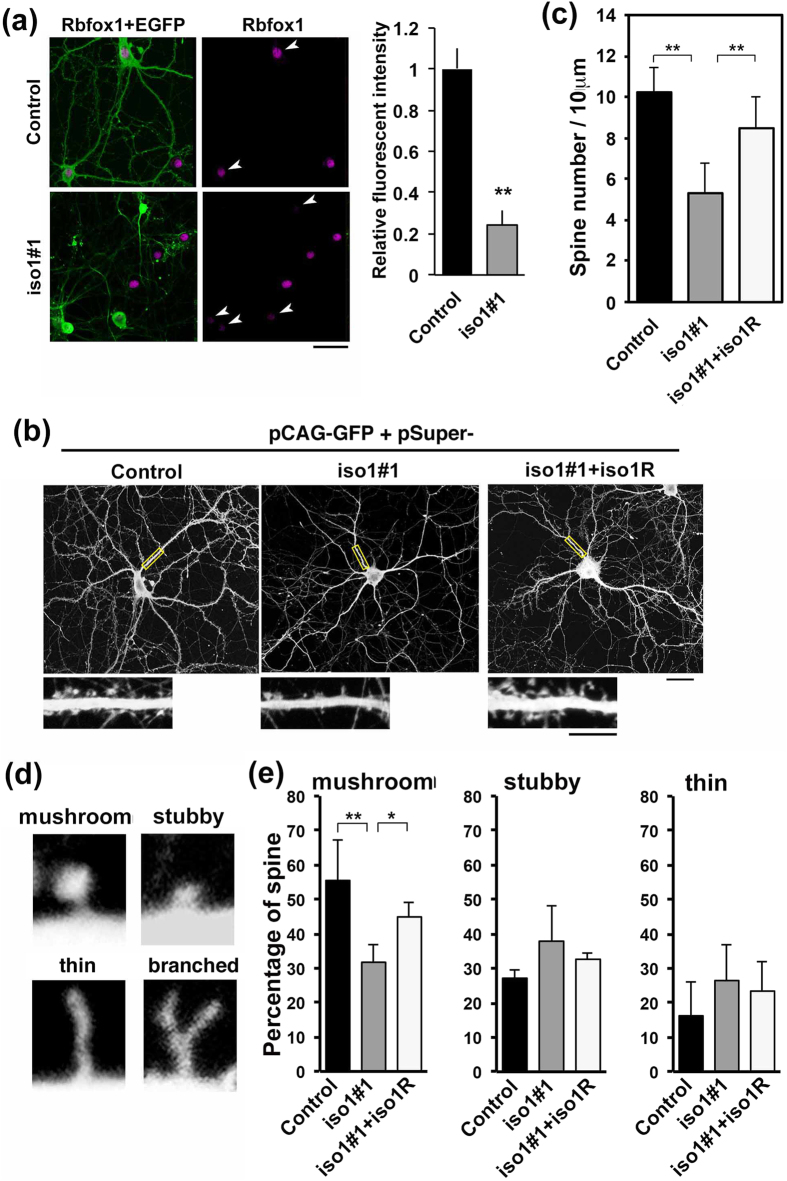
Role of *Rbfox1*-iso1 in the dendritic spine morphology in primary cultured hippocampal neurons. **(a)** Knockdown of endogenous *Rbfox1*-iso1. Neurons were transfected with pβAct-EGFP together with pSuper-H1.shLuc (Control) or pSuper-m*Rbfox1*-iso1#1 when isolated, and then fixed at 21 div. Cells were stained with anti-GFP (green) and anti-*Rbfox1* (magenta). Double-positive cells for GFP and *Rbfox1* were indicated by arrowheads. Quantification of *Rbfox1* expression was performed as in Fig. 2e. Scale bar, 20 μm. (**b**) Effects of *Rbfox1*-iso1-knockdown on the density and morphology of dendritic spines. Transfection was done as in (**a**) and stained for GFP. Rescue experiments were done with pCAG-Myc-m*Rbfox1*-iso1R. Magnified images of the indicated areas are also shown. Scale bars, 10 μm (*upper* panels) and 5 μm (*lower* panels). **(c)** Quantitative analyses of density of dendritic spines for each condition in (**b**). Error bars show SD of the results from 25–30 neurons. Experiments were repeated 3 times with similar results and representative data are shown. **p < 0.01 by Tukey-Kramer LSD. (**d**) Typical examples of mushroom, stubby, thin filopodia-like and branched spines. (**e**) Relative abundance of the spine types in neurons transfected as in (**b**). Relative percentages of each spine type were indicated. Statistical analyses were performed as in (**c**). **p < 0.01, *p < 0.05 by Tukey-Kramer LSD.

*Rbfox1*-iso1 was shown to be involved in axon-dendrite network formation. We thus further explored whether *Rbfox1*-iso1 participates in the formation of synaptic structures, since ASD and other neurodevelopmental disorders are associated with synaptic dysfunction. To this end, we assessed dendritic spine morphology in cultured mouse hippocampal neurons following *Rbfox1*-iso1-knockdown. When neurons were transfected with GFP vector together with pSuper-m*Rbfox1*-iso1#1 at 0 days *in vitro* (div) and cultured for 21 days, endogenous *Rbfox1*-iso1 was markedly silenced in the mature neurons (Fig. 9a). Under these conditions, spine density was significantly decreased in neurons transfected with pSuper-m*Rbfox1*-iso1#1 and the phenotype was rescued by m*Rbfox1*-iso1R (Fig. 9b,c). We then analyzed spine morphogenesis in *Rbfox1*-iso1-deficient neurons by counting 4 established spine morphology groups (i.e., mushroom, stubby, thin filopodia-like and branched spines) (Fig. 9d). The relative percentage of mushroom (mature) spines was significantly decreased (Fig. 9e, *left* panel). Meanwhile, the relative percentage of immature spine (stubby and thin filopodia-like spines) was increased although this was not statistically significant (Fig. 9e). The phenotype was again rescued by m*Rbfox1*-iso1R (Fig. 9e). The ratio of the branched spine was less than 2% in these assay conditions. It should be noted here that we used fixed cells in these experiments and the results are considered as snapshots at the respective time points because spine structures vary in a matter of hours.

### Electrophysiological analyses

Since *Rbfox1*-iso1 was shown to regulate synaptic structure, this molecule seems to play a pivotal role in the synaptic function, of which dysregulation may contribute to the pathogenesis of neurodevelopmental disorders. We thus examined the role of *Rbfox1*-iso1 in membrane property, spontaneous synaptic currents and NMDA (N-methyl-D-aspartate) receptor function.

**Figure 10:**
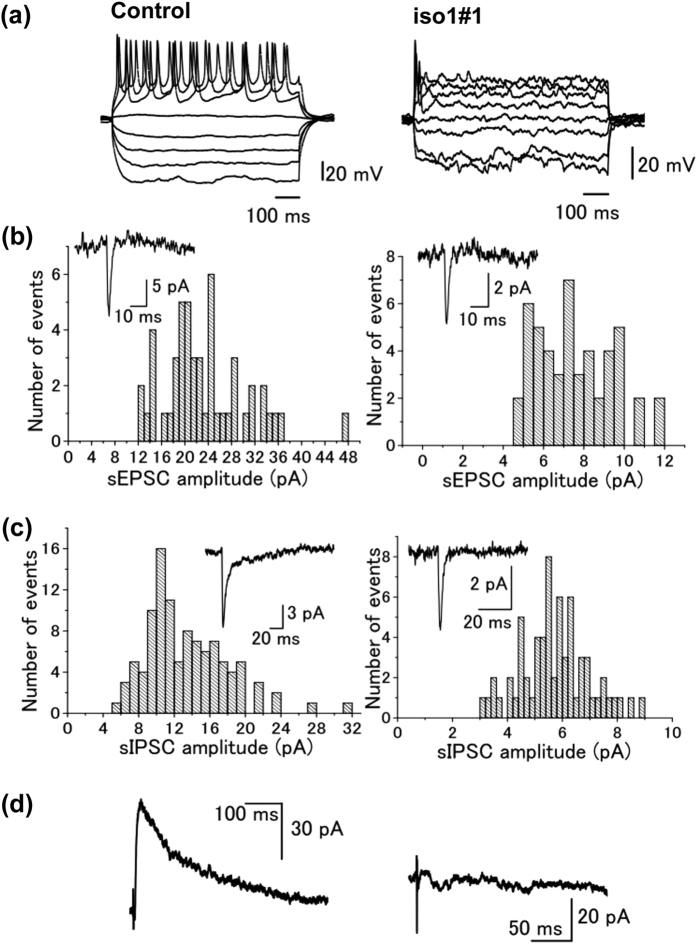
Firing and synaptic properties of control or *Rbfox1*-iso1-deficient pyramidal neurons in cerebral cortex layer II–III. **(a)** Voltage response of layer II pyramidal neurons by hyperpolarizing and depolarizing current pulses injected through recording pipettes. **(b)** Histograms showing amplitude distribution of sEPSC in layer II pyramidal neurons of control (*left* panel) and *Rbfox1*-iso-knockdown (*right* panel) mice. Inset shows the averaged sEPSC in each neuron. **(c)** Amplitude distribution of sIPSCs in control (*left*) and the knockdown (*right*) mice. Inset shows the averaged sEPSC in each neuron. **(d)** Subtracted NMDA receptor-mediated synaptic current component in control (*left*) and the knockdown (*right*) mice. Synaptic currents were evoked in the presence of bicuculline (10 μM) and strychnine (0.5 μM) at a holding potential of +40 mV. Then the NMDA receptor current components were obtained by subtracting the current in the presence of D-AP5 (25 μM) from that before D-AP5 application.

We first analyzed the effects of *Rbfox1*-iso1 on membrane properties of cortical neurons. Whole-cell recordings were made from a total of 72 layer II pyramidal neurons with fluorescence from control (n = 32) and *Rbfox1*-iso1 knockdown mice (n = 40). Both groups consist of pups of P4 or P7. Cell capacitance of neurons in control mice was 15.8 ± 0.78 pF (n = 27). On the other hand, cell capacitance in the knockdown mice was 11.4 ± 0.41 pF (n = 33), which was significantly (P < 0.05) smaller than that in control mice. Firing properties were investigated by applying hyperpolarizing and depolarizing current pulses through the recording pipette in the current-clamp mode. As shown in Fig. 10a, in control mice, the depolarizing current pulses produced multiple action potentials. Meanwhile, multiple action potentials were not produced in the knockdown mice by the same current pulses as in control mice (Fig. 10a). Such a property resembles that observed in immature neurons in the striatum^45^,^46^.

We then examined the effects of *Rbfox1*-iso1-knockdown on spontaneous synaptic currents of cortical neurons. Spontaneous synaptic currents were recorded from fluorescent layer II neurons in mice of P4. Spontaneous excitatory postsynaptic currents (sEPSCs) were recorded in the presence of bicuculline (10 μM), strychnine (0.5 μM) to block GABA_A_- and glycine receptor-mediated current component, respectively. The frequency and amplitude of sEPSCs in control mice was 0.17 ± 0.004 Hz (n = 5) and 15.8 ± 2.44 pA (n = 5), respectively (Fig. 10b,c). In the knockdown mice, sEPSC frequency was 0.16 ± 0.005 Hz (n = 8), which was not significantly (P > 0.05) different from that of control mice. On the other hand, the amplitude of sEPSCs of knockdown mice was 8.07 ± 1.31 pA (n = 8), which was significantly (P < 0.05) smaller than that of control mice. Spontaneous inhibitory postsynaptic currents (sIPSCs) were recorded in the presence of CNQX (5 μM), strychnine (0.5 μM) to block non-NMDA glutamate- and glycine receptor-mediated current component, respectively. The frequency and amplitude of sIPSCs in control mice was 0.36 ± 0.03 Hz (n = 9) and 11.6 ± 1.53 pA (n = 9), respectively (Fig. 10c). In the knockdown mice, both the frequency (0.21 ± 0.004 Hz, n = 4) and amplitude (5.86 ± 0.47 pA, n = 4) of sIPSCs were significantly (P < 0.05) smaller than the corresponding values in control mice (Fig. 10c).

Finally, the effects of *Rbfox1*-iso1-knockdown on NMDA/non-NMDA ratio of cortical neuron were analyzed with P7 mice. After making the whole-cell configuration, excitatory postsynaptic currents (EPSCs) were evoked by focal extracellular stimulation in the presence of bicuculline (10 μM) and strychnine (0.5 μM) to bock GABA_A_- and glycine receptor-mediated current components, respectively. Outward currents were evoked at the holding potential of +40 mV (Fig. 10d) at the stimulus frequency of 0.2 Hz (every 5 s). In control mice, bath application of D-AP5, an NMDA receptor blocker, at a concentration 25 μM, reduced the slow component of the outward currents. The remaining fast components were blocked by additional application of CNQX, an AMPA/kainite receptor blocker at a concentration of 5 μM, suggesting the remaining fast components are non-NMDA receptor-mediated EPSCs. NMDA-receptor mediated synaptic current components were then obtained by electrically subtracting the average of 10 consecutive currents after application of D-AP5 from those before D-AP5 application^47^. In control mice, the ratio of NMDA/non-NMDA components was 1.63 ± 0.55 (n = 4) (Fig. 10d, *left*). On the other hand, in *Rbfox1*-iso1-knockdown mice, it was generally very hard to evoke excitatory synaptic currents, probably attributable to the immature development of dendrites in the knockdown mice (Fig. 10d, *right*). Furthermore, the outward currents evoked at +40 mV were very small, and D-AP5 had only little effect on the evoked synaptic currents. Thus, obtained NMDA receptor mediated current components were virtually negligible. Such characteristics in the knockdown mice were consistently observed in all 6 neurons examined.

## Discussion

Since high frequencies in alternative splicing are thought to contribute to the functional complexity of the brain^48^, abnormalities in genes encoding neuron-specific splicing factors may induce aberrations in alternative splicing profiles in neurons, leading to the neurodevelopmental disorders. While 5 isoforms of *RBFOX1* have been reported in human, 7 isoforms have been identified in mice^1^,^23^,^49^. Recently, we analyzed the pathophysiological significance of *Rbfox1*-isoform5 (A2BP1-A30)^22^. In this study, we examined the role of *Rbfox1*-iso1 in the cortical lamination and synapse network formation. The primary stuctures of the 2 isoforms are different in their C-termini. The C-termnal region of *Rbfox1*-iso1 is 66 aa in length, whereas this is replaced with a 43 aa sequence in *Rbfox1*-iso5. The results of this study support the notion that disrupted *RBFOX1*-iso1 function accounts for the emergence of the clinical symptoms in neurodevelopmental and psychiatric disorders caused by *RBFOX1* gene abnormalities.

Central nervous system-specific *Rbfox1*-knockout mice, in which both nuclear and cytoplasmic isoforms are null, exhibited susceptibility to seizures and increase in neuronal excitability in the dentate gyrus but did not show gross morphological alteration in cerebral cortex^6^. It is notable that Rbfox2 expression was increased in the knockout mice^6^ whereas its expression in *Rbfox1*-iso1-knockdown neurons was comparable to that in control cells. Acute transfer of RNAi vector *in utero* may avoid the changes of Rbfox2 expression observed in the knockout mice. We assume that the apparently mild phenotype observed in *Rbfox1*-knockout mice may be due to the compensatory effects by amplified Rbfox2^6^. It is our contention that acute knockdown by *in utero* electroporation may circumvent the compensatory effects. For example, knockout mice for *Sept4* (Parkinson disease-related protein) and *SIL1*-deficient mice (a model for Marinesco-Sjogren syndrome) exhibited little architectural alteration in the cerebral cortex^50^,^51^ whereas acute knockdown of these genes induced defects in neuronal migration^31^,^52^. Alternatively, the phenotype of *Rbfox1*-iso1-deficient neurons in this study could be highlighted in surrounding normal neurons since the deficient cells are present among non-transfected normal neurons while the neurons in the knockout mice are completely surrounded with the same *Rbfox1*-null cells. Meanwhile, we found decrease in spine density in *Rbfox1*-iso1-deficient hippocampal neurons *in vitro*, which was consistent with the phenotype of the knockout mouse^6^. Rbfox2 protein expression was reported to increase in acute *Rbfox1*-knockdown experiments with isolated hippocampal neurons^53^. Collectively, experimental conditions such as the cell environment and culture conditions used to suppress *Rbfox1* expression may differently influence the Rbfox2 expression.

Electrophysiological analyses demonstrated significant changes in membrane and synaptic properties in knockdown mice when compared to the control. The membrane properties of the knockdown mice were similar to those of immature neurons in the developing or regenerated neurons in the striatum^45^,^46^. Less frequency and smaller amplitude of both excitatory and inhibitory synaptic currents could be attributable to the reduced development of dendrites observed in the morphological studies. The reduced NMDA/AMPA ratio of evoked excitatory synaptic currents in the knockdown mice, as well as the abnormality in neuronal migration and morphology, is in agreement with a previous study showing that NMDA receptors are involved in neuronal migration and morphological changes into a bipolar shape^34^.

To elucidate the pathophysiological significance of *RBFOX1* in ASD and other neurodevelopmental disorders, identification of *RBFOX1* target molecules will be essential. Since neuronal migration requires the orchestrated remodelling of the cytoskeleton^54^,^55^, it is reasonable that genes important for cytoskeleton reorganization are among *RBFOX1* target molecules^6^. Based on the results obtained here, *RBFOX1* is most likely to regulate the radial migration and terminal translocation through the regulation of nucleokinesis, in which microtubules and the centrosome play central roles. In this context, a microtubule-binding protein, Doublecortin, is a candidate target for *RBFOX1*^56^. Another target molecule candidate, FilaminA (an actin-binding protein), is also involved in cortical neuron migration^57^. It is tempting to speculate that Doublecortin and/or FilaminA are downstream targets for *Rbfox1* and regulate cortical neuron migration as well as spine morphology. In addition, transcriptome analyses revealed neurologically relevant genes such as *SLC1A3, DCLK1, GABRB3, GAD2, KCNQ2, SCN8A, SLC12A5, DBN1, NLGN3, NLGN4X, SV2B* and *SYN1* as the spicing targets for *Rbfox1*^7^,^56^,^58^. These are known to be causal or candidate genes for various neurodevelopmental disorders such as ID, ASD and epilepsy. Further intensive analysis of the molecular machinery downstream of *RBFOX1*-mediated splicing should contribute to a better understanding of the mechanisms of neurodevelopmental and psychiatric disorders where *RBFOX1* abnormalities are involved.

In this report, we focused on the pathophysiological relevance of *Rbfox1*-iso1. When the knockdown phenotypes were compared to those of cytosolic minor isoform, m*Rbfox1*-iso5, inhibitory effects by m*Rbfox1*-iso1-knockdown on the radial migration and axon elongation to the contralateral cortex were stronger under the same assay conditions^22^. Also, while the number of stubby-shaped immature spines increased when m*Rbfox1*-iso5 was silenced in primary cultured hippocampal neurons^22^, filopodia-like immature spines in addition to stubby spines increased in *Rbfox1*-iso1-deficient cells. Overall, similar but not identical results were obtained in the knockdown phenotypes of the 2 isoforms. This similarity and difference in the phenotypes might be explained by distinct roles of nuclear and cytoplasmic *Rbfox1* isoforms in convergent signaling pathways during cortical development. Interestingly, a recent study revealed different molecular functions of nuclear and cytoplasmic *Rbfox1* isoforms. Matrin and colleagues clarified that nuclear *Rbfox1* is involved in splicing changes while cytoplasmic *Rbfox1* regulates the stability and translation of the target mRNAs^53^. As mentioned earlier, many *Rbfox1* target candidates take part in cortical development and neurodevelopmental disorders including ASD. Although such candidate proteins are possible to be processed differently at different intracellular sites by *Rbfox1* isoforms, signaling pathways where these molecules are involved may converge to regulate corticogenesis in a coordinate manner.

*Rbfox1*-iso1-deficiency caused defects in neuronal cell morphology, migration, synapse network formation and synapse physiology during brain development. The obtained results elucidated essential roles of *Rbfox1*-iso1 in brain development, and support the hypothesis that functional defects of *RBFOX1*-iso1 may be related to the etiologies of neurodevelopmental disorders. As for an underlying mechanism in the migration defects, disrupted nucleokinesis caused by aberrant microtubule-centrosome interaction is considered to be a core event. While abnormal nucleokinesis causes hampered radial migration and terminal translocation, multipolar movement was apparently normal in *Rbfox1*-deficient cells, perhaps due to the crucial role of actin cytoskeleton in this movement. Abnormal actin reorganization is supposed to be responsible for the aberrant spine morphology, and indeed various actin-related molecules are target candidates for *Rbfox1*. Although disrupted spine function is thought to play an important role in pathophysiology of ASD and other neurodevelopmental disorders, underlying molecular mechanism(s) remains to be elucidated.

## Conclusions

With a sophisticated system biology analyses, *RBFOX1* was recently shown to serve as a “hub” in ASD-gene transcriptome networks, strongly suggesting its crucial role in the pathophysiology of ASD. Since *RBFOX1* gene abnormalities have also been found in other neurodevelopmental and psychiatric disorders including ID, ADHD and schizophrenia, this gene product is supposed to have an essential role in neuronal function and corticogenesis.

To elucidate the pathophysiological relevance of *RBFOX1*, we here focused on the dominant neuronal isoform of *Rbfox1* (*Rbfox1*-iso1) localized in the nucleus and examined its role during mouse corticogenesis *in vivo* and *in vitro.* Knockdown of *Rbfox1*-iso1 *in vivo* caused defects in the radial migration and terminal translocation of cortical neurons. *Rbfox1*-iso1 was also involved in the synapse network formation and/or maintenance through the regulation of axon growth and dendritic arborization *in vivo*. In addition, electrophysiology analyses revealed significant defects in membrane and synaptic properties in *Rbfox1*-iso1-deficient neurons, indicating that *Rbfox1*-iso1 is essential for synapse functions. Further *in vitro* experiments revealed reduction of spine density and mature spine number in *Rbfox1*-iso1-deficient mouse hippocampal neurons. The abnormal phenotypes observed were similar but not the same as those in the deficient neurons of cytoplasmic minor isoform, *Rbfox1*-iso5.

In summary, we report an essential role of *Rbfox1*-iso1 in cortical development. Impairment of *Rbfox1*-iso1 function may induce structural and functional defects of the cerebral cortex, and consequently contribute to the clinical symptoms of ASD and other neurodevelopmental disorders with *RBFOX1* gene abnormalities.

## Additional Information

**How to cite this article**: Hamada, N. *et al*. Essential role of the nuclear isoform of *RBFOX1*, a candidate gene for autism spectrum disorders, in the brain development. *Sci. Rep.* **6**, 30805; doi: 10.1038/srep30805 (2016).

## Supplementary Material

Supplementary video 1

Supplementary video 2

Supplementary video 3

Supplementary video 4

Supplementary video 5

Supplementary video 6

Supplementary Information
