# Supplementary material for: Essential role of the nuclear isoform of RBFOX1, a candidate gene for autism spectrum disorders, in the brain development
Source: Sci Rep. 2016 Aug 2;6:30805. doi: 10.1038/srep30805 (PMC4969621; doi:10.1038/srep30805)
Supplement: Supplementary Information [file srep30805-s7.pdf]

Supplementary Information

Essential role of the nuclear isoform of *RBFOX1*, a candidate gene for autism spectrum disorders, in the brain development

Nanako Hamada[1,2], Hidenori Ito[1], Takuma Nishijo[3], Ikuko Iwamoto[1], Rika Morishita[1], Hidenori Tabata[1], Toshihiko Momiyama[3] and Koh-ichi Nagata[1,4]

[1]Department of Molecular Neurobiology, Institute for Developmental Research, Aichi Human Service Center, Kasugai; [2]Research Fellow of Japan Society for the Promotion of Science; [3]Department of Pharmacology, Jikei University School of Medicine, Tokyo; [4]Department of Neurochemistry, Nagoya University Graduate School of Medicine, Nagoya, Japan

Supplementary Videos

Supplementary video 1: Time-lapse imaging of morphological change of control neurons migrating in upper IZ - lower CP.

Supplementary video 2: Time-lapse imaging of morphological change of Rbfox1-iso1-defcient neurons stranded in upper IZ - lower CP.

Supplementary video 3: Time-lapse imaging of morphological change of control neurons migrating in CP.

Supplementary video 4: Time-lapse imaging of morphological change of Rbfox1-iso1-defcient neurons migrating in CP.

Supplementary video 5: Time-lapse imaging of N-C distance dynamics of a control neuron migrating in CP.
Yellow and white arrowheads indicate centrosome and nucleus, respectively.

Supplementary video 6: Time-lapse imaging of N-C distance dynamics of an Rbfox1-iso1-defcient neuron migrating in CP.
Yellow and white arrowheads indicate centrosome and nucleus, respectively.
